# Estimation of Mechanical and Transport Parameters in Cancers Using Short Time Poroelastography

**DOI:** 10.1109/JTEHM.2022.3198316

**Published:** 2022-08-16

**Authors:** Sharmin Majumder, Md Tauhidul Islam, Raffaella Righetti

**Affiliations:** Department of Electrical and Computer EngineeringTexas A&M University College Station TX 77843 USA; Department of Radiation OncologyStanford University 6429 Stanford CA 94305 USA

**Keywords:** Cancer imaging, poroelastography, strain time constant, vascular permeability, Young’s modulus and Poisson’s ratio

## Abstract

Mechanical and transport properties of cancers such as Young’s modulus (YM), Poisson’s ratio (PR), and vascular permeability (VP) have great clinical importance in cancer diagnosis, prognosis, and treatment. However, non-invasive estimation of these parameters *in vivo* is challenged by many practical factors. Elasticity imaging methods, such as “poroelastography”, require prolonged data acquisition, which can limit their clinical applicability. In this paper, we investigate a new method to perform poroelastography experiments, which results in shorter temporal acquisition windows. This method is referred to as “short-time poroelastography” (STPE). Finite element (FE) and ultrasound simulations demonstrate that, using STPE, it is possible to accurately estimate YM, PR (within 10% error) using windows of observation (WoOs) of length as short as 1 underlying strain Time Constant (TC). The error was found to be almost negligible (< 3%) when using WoOs longer than 2 strain TCs. In the case of VP estimation, WoOs of at least 2 strain TCs are required to obtain an error < 8% (in simulations). The stricter requirement for the estimation of VP with respect to YM and PR is due its reliance on the transient strain behavior while YM and PR depend on the steady state strain values only. *In vivo* experimental data are used as a proof-of-principle of the potential applicability of the proposed methodology *in vivo*. The use of STPE may provide a means to efficiently perform poroelastography experiments without compromising the accuracy of the estimated tissue properties.

## I. Introduction

Pathological changes typically alter the mechanical properties of tissues [1], [2]. To describe the mechanical behavior of linear elastic tissues, at least two parameters are required - namely the Young’s modulus (YM) and the Poisson’s ratio (PR). YM is a mechanical parameter that can be used as a measure of the stiffness of a tissue. In homogeneous linear elastic solids, the YM is defined as the ratio between the applied axial stress and the resulting axial strain. In inhomogeneous solids, the YM can be obtained by dividing the local axial stress developed inside the solid due to the applied stress by the resulting local axial strain [3]. PR provides a measure of the compressibility of a solid. In homogeneous linear elastic solids (under uniaxial compression), the PR is defined as the negative ratio between the lateral strain and the axial strain. In inhomogeneous solids, the PR is related to the lateral to axial strain ratio in a more complex manner [3].

While many studies reported in the literature assume that biological tissues behave as linear elastic solids, there is a growing body of evidence indicating that poroelastic models may provide a more realistic description of the mechanical behavior of complex tissues than linear elastic models [4], [5]. A poroelastic material is, by definition, compressible [6]. Immediately after the application of a uniaxial stress, a poroelastic tissue behaves as an incompressible solid with effective PR close to 0.5. Then, relaxation takes place inside the tissue, dynamic processes occur, which are accompanied by changes in the spatial and temporal distributions of the strains. In steady state conditions, the tissue behaves as a linear elastic solid. Therefore, YM and PR can be used to quantify the stiffness and compressibility of a poroelastic tissue, as long as their measurements are computed at steady state [6].

Estimation of vascular permeability (VP) may be important for cancer prognosis [7] and may be useful for the choice of a cancer treatment [8], [9]. Moreover, VP affects the values of convection and consolidation time of drug molecules [5]. Therefore, it may be an important parameter for drug delivery therapies.

There are only a few non-invasive imaging modalities that can generate YM maps of tissues *in vivo*. Ultrasound strain elastography [10], ultrasound shear wave elastography (SWE) [11] and magnetic resonance elastography (MRE) [12] techniques have shown to be able to provide YM images, under the assumption that the tissue behaves as a linearly elastic incompressible solid [13], [14], [15], [16], [17], [18]. In a recent study, we have proposed a new method to assess the YM based on the poroelastic assumption of tissues [6]. This method uses Eshelby’s formulation to theoretically estimate both the YM and the PR of tissues, and, therefore, it does not assume incompressibility [3]. We demonstrated that our methods can accurately estimate YM and PR in tumors of different shape and size and under different boundary conditions. To our knowledge, this is the only method retrievable in the literature to non-invasively image the actual PR in non-homogeneous tissues *in vivo*. There are currently a few techniques that can be used to image VP in soft tissues. Among the invasive techniques, the methods proposed in [19], [20] are the most commonly employed ones. Non-invasive techniques for estimating the VP in tumors and normal tissues have also been explored [7], [21], [22], [23].

Ultrasound strain elastography (USE) is a popular, cost-effective and non-invasive imaging modality that can be used to assess the strains generated in a tissue in response to a small compression [10], [24]. Poroelastography is an elastographic technique that aims at estimating the response of poroelastic tissues upon sustained compression [25], [26], [27]. In a poroelastography experiment, the tissue is compressed for a certain time interval while a series of radio frequency (RF) data is acquired, from which time-dependent axial and lateral strain elastograms are generated. The temporal and spatial distributions of the axial and lateral strains carry information about fluid transport parameters such as VP and interstitial permeability (IP) [28]. The steady state strains can be used to evaluate the tissue mechanical properties.

Estimation of YM, PR and VP using poroelastography requires prolonged experimental data acquisitions (typically > 1 minute). These prolonged experiments can introduce motion-noise in data, which can result in erroneous values for the estimated parameters. In a previous paper [29], we performed a simulation study to investigate the shortest windows of observation (WoOs) to achieve accurate strain time constant (TC) estimation. One-dimensional (1D) simulation demonstrated that WoOs of at least 20% of the underlying strain TC are needed to obtain strain TC estimations with error < 10%, assuming very fast acquisition rates (>100 Hz). Phantom experimental data, however, showed the need of WoOs longer than those predicted by simulations (>50% the underlying strain TC). The requirement may be stricter for short underlying strain TC [29]. This study was performed using 1D simulations and did not investigate the effect of reduced length WoOs on the reconstructed mechanical or transport parameters. Moreover, the 1D simulation data were created from the strain-vs-time curve assuming very high acquisition rates that may be unrealistic in ultrasound poroelastography experiments.

In this paper, we demonstrate, both theoretically and experimentally, that it is possible to accurately assess YM, PR and VP in a cancer animal model using reduced length WoOs. This new poroelastographic acquisition method is referred to as “short time poroelastography” (STPE). This study and related findings can be beneficial for future planning of clinical poroelastography applications.

## II. Methods

### A. Problem Formulation

**FIGURE 1.**
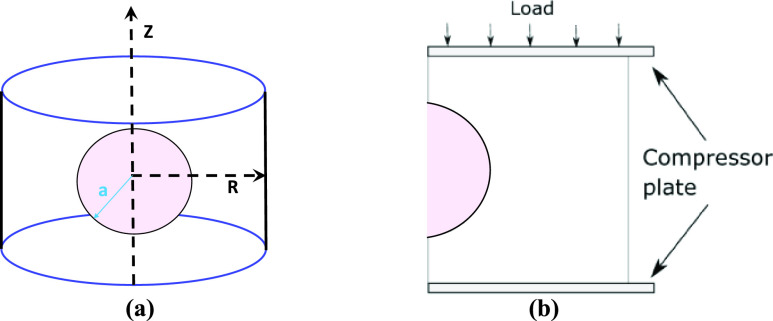
A schematic of a cylindrical sample of a poroelastic material with a spherical inclusion of radius a. The axial direction is along the z axis. “R” refers to the radial direction in the cylindrical coordinate system. (b): The 2D solution plane for the 3D sample in (a). The sample is compressed between two compressor plates. The compression is assumed to be uniaxial.

For the theoretical formulation of the problem, we assume spherical/elliptical inclusions embedded in a cylindrical background [6], [30]. Our theoretical models are inherently three-dimensional (3D) [6]. Our simulation model was developed based on the assumption of axisymmetric conditions [6], [30]. A schematic diagram of a cylindrical sample with a spherical inclusion under creep compression is shown in Fig. 1.

The temporal behavior of the strains (first order exponential approximation) in a poroelastic sample during creep compression, can be described using as a generalized model [30], [31]

\begin{equation*} s\left ({t }\right)=\eta +\left ({\alpha -\eta }\right)e^{-\frac {t}{\tau }}\tag{1}\end{equation*}

where $s\left ({t }\right)$ is the value of the strain (axial/lateral) at time t, $\alpha $ is the strain at time $0^{+}$ (i.e., immediately after the application of the excitation), $\eta $ is the strain at steady state (i.e., at $\infty $ time), and $\tau $ is the TC of the strain.

The applied strain in elastography experiments is typically very small (1% - 5%) since decorrelation errors increase with large tissue strain values [32]. Therefore, in typical poroelastography creep compression experiments, the absolute difference between the axial strain at $\text{t}= 0^{+}$ ( $\alpha$) and the axial strain at $\text{t}= \infty $ ( $\eta$) is < 10%, i.e., $\left ({\eta -\alpha }\right){ < 0.1}$. For the lateral strain, that difference may be smaller. Assuming $s\left ({t }\right)$ in equation (1) to be the axial strain and without loss of generality assuming that $\left ({\eta -\alpha }\right) < t^{\prime }$, then equation (1) can be written as,

\begin{equation*} s\left ({t }\right) > \eta -t'e^{-\frac {t}{\tau }}\tag{2}\end{equation*}

If the strain data are acquired for a time $t=2\tau $, $t=1\tau $, and $t=0.5\tau $, then the axial strain will be $s\left ({t }\right) > \eta -0.135t'$, $s\left ({t }\right) > \eta -0.368t'$, and $s\left ({t }\right) > \eta -0.6t'$, respectively.

As we mentioned earlier, according to strain filter theory [32], during poroelastography experiments, axial strain is not usually not more than 10%. If it is 10% then $t' =0.1$, $s\left ({t }\right)\mathrm { > 0.98}~\eta,s\left ({t }\right)\mathrm { > 0.96}~\eta $, and $s\left ({t }\right) > 0.94~\eta $ for $t=2\,\,\tau,t=1\,\,\tau $ and $t=0.5\,\,\tau $ (as $0.5~\tau $ was considered by our previous empirical study [29]), respectively. Moreover, even if the axial strain is more than 10% (let’s say 15%) then $t' =0.15$, then we can still write $s\left ({t }\right)\approx 0.98~\eta $ for $t=2\tau $. In the above cases, the difference between the estimated strains at different time points considered and the steady state are negligible. Therefore, theoretically, the reconstructed YM and PR should have a negligible error as most of the strain information is available for their estimations. With respect to the WoOs requirements for accurate estimation of VP, the conditions may be different as VP requires knowledge of the YM, PR as well as the strain TC.

### B. Determination of Axial and Lateral Strain

To compute axial and lateral displacements from the pre- and post-compressed RF data, we used a two-step processing method, referred as “DPHS”, proposed in [33]. “DPHS” stands for Dynamic Programming Elastography (DPE) and Horn-Shunk (HS) optical flow estimation methods. DPE is first used to obtain the integer axial and lateral displacement estimates [53]. This step is then followed by a motion compensation procedure and HS optical flow estimation to obtain subsample estimates of both the axial and lateral displacements. In DPE, displacement continuity is assumed based on which a regularized cost function is created. Based on the cost function minimization, integer axial and lateral displacements are computed. Integer axial and lateral displacement values obtained from DPE and residual sub-pixel axial and lateral displacement values obtained from the HS method are added to obtain the complete axial and lateral displacement estimations. Considering both integer and sub-sample displacements improves the quality of axial and lateral displacements. Axial and lateral strains are then calculated from the axial and lateral displacements using Kalman filter-based least square estimation [33].

### C. Determination of YM and PR

We simulated tumors of two shapes: spherical and elliptical. To determine the YM and PR of the tumor (inclusion) and background tissue, we use the theories developed in [6]. In brief, the YM and PR of the tumor are estimated using a cost function minimization technique developed for the Eshelby’s theory in case of inclusion embedded in a background. The cost function is the squared value of the difference between two expressions of the same eigen function inside the inclusion developed by Eshelby and Mura in [3]. One expression of the eigen function incorporates the YM and PR of both the tumor and normal tissue, and the Eshelby tensor. The other expression of eigen strain incorporates only the Eshelby tensor. Both expressions use the axial and lateral strains at steady state from the poroelastography experiment.

In this proposed approach, the Eshelby tensor depends on the geometry of the inclusion and the PR of the background [6]. Therefore, Eshelby tensor changes if the inclusion’s shape changes. We have determined the Eshelby tensor for both elliptical and spherical inclusions as detailed in the Supplementary of [6]. Thus, our developed model for STPE is applicable to both spherical and elliptical tumors with a change in the parameter computation procedure.

In case of the *in vivo* experiment, the tumor shape is typically not regular. Therefore, we approximated the tumor with its best fit ellipsoid using eigen decomposition [30] and then used the Eshelby tensor developed for elliptical inclusions to reconstruct YM and PR inside the inclusion in the *in vivo* data.

### D. Determination of VP

**FIGURE 2.**
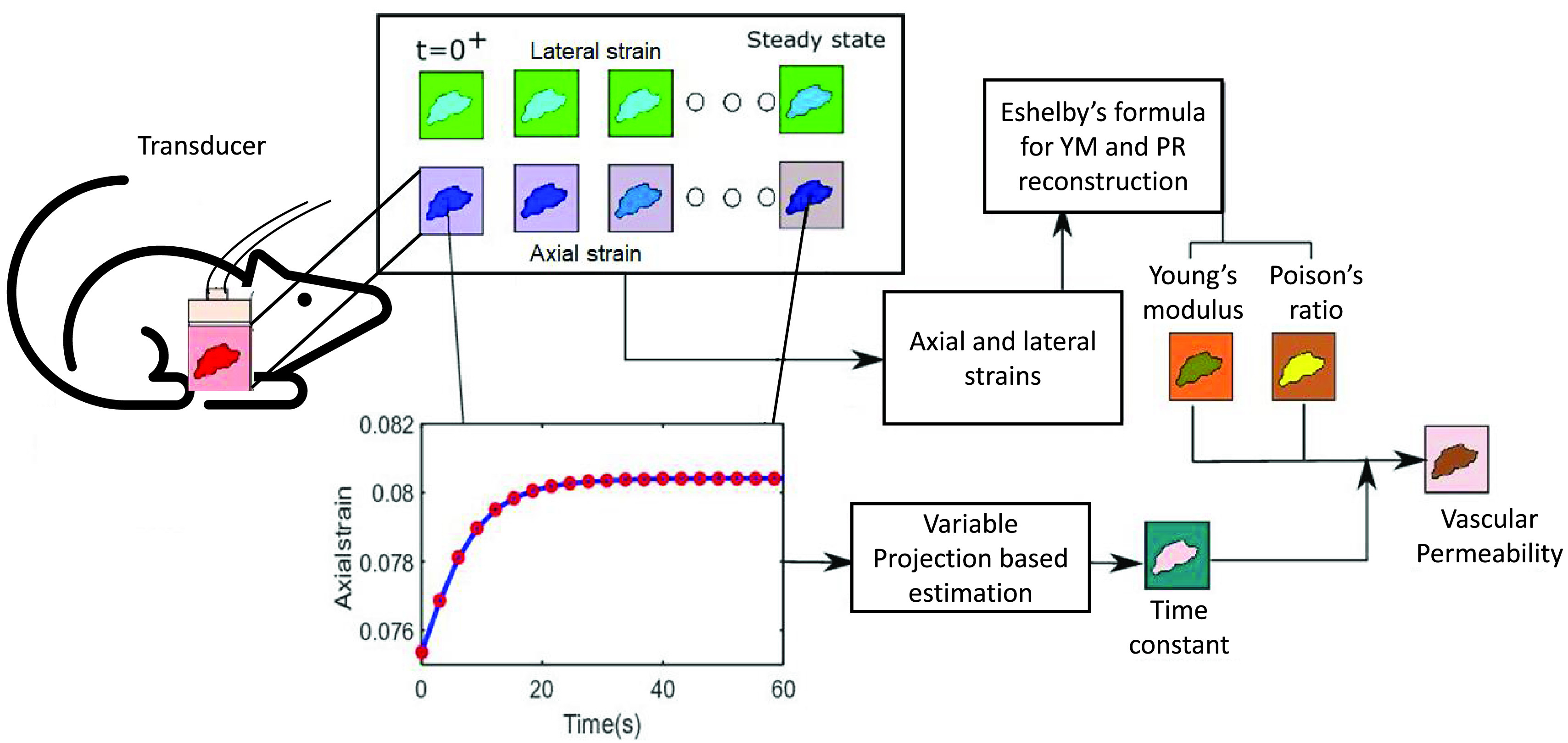
Block diagram to estimate YM, PR and VP in cancer in vivo. RF data is captured by the ultrasound transducer. Spatial and temporal axial and lateral strains are computed from the RF data. From the steady state axial and lateral strains, YM and PR are computed using Eshelby’s theory. Axial strain time constant (TC) is computed from the temporal axial strain by variable projection method. Vascular permeability (VP) is computed by knowledge of YM, PR, and TC. In our proposed short time poroelastography method, axial and lateral strains data up to 2 TC (~20s) are used.

A block diagram for our proposed technique to estimate VP is shown in Fig. 2. Technical details of this technique can be found in [30].

For the spherical model shown in Fig. 1, the microfiltration coefficient is given by $\chi =L_{p}S \mathord {\left /{ {\vphantom {S V}} }\right. } V$, where *Lp* is the hydraulic conductivity (also referred to as “VP”) and $S \mathord {\left /{ {\vphantom {S V}} }\right. } V$ is the surface area to volume ratio of capillary walls inside the sample. Assuming $\chi \gg k \mathord {\left /{ {\vphantom {k a^{2}}} }\right. } a^{2}$, where $k$ is the interstitial permeability and $a$ is the radius of the inclusion, we can write the axial strain TC as $\tau =1/(H_{A}\chi)$ [30]. $H_{A}$ is aggregate modulus and is defined as $H_{A}=E(1-\nu)/(1-2\nu)(1+\nu)$ where $E$ and $\nu $ are the YM and PR, respectively. Note that this TC expression is applicable to the strains in both the inclusion and the background as long as $\chi \gg k \mathord {\left /{ {\vphantom {k a^{2}}} }\right. } a^{2}$ is valid. However, this assumption may not be valid in the normal tissue (background) [30]. By knowledge of the axial strain TC, YM and PR, VP can be estimated. To estimate the axial strain TC, temporal strain data are first denoised by an empirical orthogonal function (EOF) analysis method [36] and fitted with equation (1) using a variable projection technique [37]. It is noted that our variable projection curve fitting method provides estimates of both the strain TC and the strain steady state value ( $\eta$). To compute YM and PR, we use the steady state axial and lateral strains predicted by the curve fitting estimator for shorter WoOs (0.5 TC, 1 TC, 2 TC) as well as the full data length. In the estimated axial strain TC map, outliers in the inclusion (i.e., pixels with value $ > 100\text{s}$) were replaced with the median value of the strain TC inside the inclusion.

## III. Simulations

### A. FE Simulation

The commercial FE simulation software ABAQUS (Abaqus Inc, Providence, RI, USA) [38] was used to create the displacements and strains in the simulated poroelastic samples used in this study (under boundary conditions mimicking the real USE experiments). An ‘effective stress’ principle is used in ABAQUS, where the total applied stress is assumed to be sum of the fluid stress and the elastic or effective stress [38].

A schematic of the geometry of the simulated phantoms is shown in Fig. 1. The poroelastic samples were modeled as linearly elastic, isotropic, incompressible, permeable solid phases saturated with an incompressible fluid. The samples were compressed from the top and the bottom side was kept static. For each sample, a single 2D solution plane was analyzed in ABAQUS. The mesh element type used for this analysis was CAX4RP and there were 24,530-24,900 elements in the solution plane. The properties of the element type in CAX4RP are 4-node bilinear displacement and pore pressure, reduced integration with hourglass control [38]. The dimension of the solution plane for all the simulated samples is 20 mm (lateral dimension) and 40 mm (axial dimension). Zero fluid pressure boundary conditions on the right-hand side of the samples were imposed to ensure the flow of the fluid only in the right direction. An instantaneous stress of 1 kPa was applied for creep experiment simulation.

Table 1 summarizes the simulation cases analyzed in this study. In samples A, B, C, and D, we assumed the inclusion to be spherical (7.5 mm diameter), whereas, in samples E and F, we assumed the inclusion to be elliptical with 7.5 mm length along lateral direction and 5 mm along the axial direction. Table 1 shows also the true mechanical and transport parameters used for our simulations. These parameters were chosen following prior studies [5], [39]. In Table 1, subscript $i$ denotes the inclusion and $b$ denotes the background. In all cases, $S/V$ was assumed to be 20,000 m^−1^ for both the tumor and normal tissue as indicated in [40]. The total analysis was recorded for 60s with a temporal resolution of 0.1s for samples A, B, C, E, and F. For sample D, the data recording time was considered 120s with a temporal resolution of 0.2s (as the inclusion strain TC of sample D is > than the one for the other samples).

**TABLE 1:** Description of the Poroelastic Samples. $E$: Young’s Modulus, $\nu$: Poisson’s Ratio, $L_{p}$: Vascular Permeability, $k$: Interstitial Permeability, $\tau$: Time Constant. Subscripts $i$ Denotes the Inclusion and $b$ Denotes the Background

Inclusion shape	Sample name	$E_{i}$ (kPa)	$\nu _{i}$	$L_{p,i}$ (m(Pa $\cdot \text{s}$)^−1^)	ki ( $\text{m}^{4}\text{N}^{-1}\text{s}^{-1}$)	$E_{b}$ (kPa)	$\nu _{b}$	$L_{p,b}$ (m(Pa $\cdot \text{s}$)^−1^)	$k_{b} $( $\text{m}^{4}\text{N}^{-1}\text{s}^{-1}$)	$\tau _{i}$ (s)	$\tau _{b}$ (s)
Spherical	A	50	0.45	$2.82\times 10 ^{-11}$	$3.1\times 10 ^{-14}$	32.78	0.47	$1.89\times 10 ^{-11}$	$6.1\times 10 ^{-15}$	9.348	13.43
	B	97.02	0.45	$2.82\times 10 ^{-11}$	$3.1\times 10 ^{-14}$	32.78	0.47	$1.89\times 10 ^{-11}$	$6.1\times 10 ^{-15}$	4.818	13.43
	C	97.02	0.4	$2.82\times 10 ^{-11}$	$3.1\times 10 ^{-14}$	32.78	0.47	$1.89\times 10 ^{-11}$	$6.1\times 10 ^{-15}$	8.52	13.43
	D	97.02	0.47	$2.83\times 10 ^{12}$	$3.1\times 10 ^{-14}$	32.78	0.49	$9.45\times 10 ^{-13}$	$6.1\times 10 ^{-15}$	30.25	90.43
Elliptical	E	97.02	0.4	$2.82\times 10 ^{-11}$	$3.1\times 10 ^{-14}$	32.78	0.47	$1.89\times 10 ^{-11}$	$6.1\times 10 ^{-15}$	8.52	13.43
	F	50	0.45	$5.65\times 10 ^{-11}$	$3.1\times 10 ^{-14}$	32.78	0.47	$1.89\times 10 ^{-11}$	$6.1\times 10 ^{-15}$	4.66	13.43

### B. Ultrasound Simulation

To investigate the effect of noise and ultrasonic limitations on the performance of STPE, simulated pre- and post-compression temporal ultrasound radio frequency (RF) data were generated from the mechanical displacements obtained from FE simulation using a convolution model [41]. In this model, the object field is convolved with the point spread function (PSF) of the transducer to obtain the transducer response. A Gaussian noise of specific SNR, which depends on the measurement and electronic noise, is then added to the data to obtain the pre-compressed RF data. The object field consists of randomly distributed speckle. Bilinear interpolation is performed on the pre-compressed object field using the pre- and post-compressed coordinates of the speckles to obtain the post-compressed object field. Similar to pre-compressed RF data, post-compressed RF data is obtained by convolving the post-compressed object field with the PSF and then adding noise of specific SNR. We simulated a 38-mm linear array transducer (Sonix RP, Ultrasonix, Richmond, BC, Canada) with a center frequency of 6.6 MHz and 50% fractional bandwidth (at −6 dB). The simulated ultrasound transducer resembled our experimental transducer having the same ultrasonic properties. The transducer’s beamwidth was assumed to be dependent on the wavelength and to be approximately 1 mm at the focus [27]. The sampling frequency was set at 40 MHz. We simulated pre- and post- compressed RF data with Gaussian noise of SNR 20 dB, or 40 dB. To compute the elastograms from the pre- and post-compressed RF data, we used Dynamic programming and Horn-Schunk (DPHS) method described in [33].

### C. Image Quality Analysis for Simulated Data

Quality of YM/PR/VP images was quantified using percent relative error (PRE). We considered 3 scenarios: a) WoO = 2 TC; b) WoO = 1 TC; and c) WoO = 0.5 TC. For each case, the PRE was computed between the estimated parameters using the WoO and the steady state strain data. PRE is defined as [37]

\begin{align*} PRE=\sum \nolimits _{c=1}^{C} \sum \nolimits _{r=1}^{R} {\frac {abs\left ({\rho _{f\left ({r,c }\right)}-\rho _{s}\left ({r,c }\right) }\right)}{\rho _{f\left ({r,c }\right)}}\times \frac {100}{R\times C}} \\\tag{3}\end{align*}

where $\rho _{f}$ is the estimated parameter using the full duration strain data (i.e., steady state) and $\rho _{s}$ is the estimated parameter using the WoO. $R$ and $C$ represent the row and column in the estimated YM/PR/VP maps, respectively. The PRE analysis is performed only for the inclusion.

## IV. Experiments

As a proof of principle of the applicability of STPE *in vivo*, we report results obtained from two mice with triple negative breast cancer cells injected in the mammary fat pad [42]. The cancer was created at the Houston Methodist Research Institute. *In vivo* data acquisition was approved by the Houston Methodist Research Institute, Institutional Animal Care and Use Committee (ACUC-approved protocol # AUP-0614-0033). Prior to ultrasound data acquisition, the mouse was anesthetized with isoflurane. Data acquisition session was 1 minute long. The sampling period of the data was set at 0.1 s.

Elastography was carried out using a 38-mm linear array transducer (Sonix RP, Ultrasonix, Richmond, BC, Canada) with a center frequency of 6.6 MHz and 5–14 MHz bandwidth. To compensate for the surface geometry as well as facilitate positioning the focus inside the superficial tumors, an aqueous ultrasound gel pad (Aquaflex, Parker Laboratories, NJ, USA) was placed between the compressor plate and the developed tumor. Creep compression was performed on the animal with the duration of compression being one minute [6]. Each experiment was repeated for 5-6 times [6].

A force sensor (Tekscan FlexiForce, manufactured by Tekscan, Inc., South Boston, MA, USA-02127) was inserted between the gel pad’s top surface and the compressor plate to record the applied force during the compression. A Microsoft Windows based interface software is provided with the sensor and can be used to observe and record the applied force. The sensor used in the kit is model #A201, which senses a force range $0-4.4$ N in a scale of 0–255. The diameter of the sensing area of the sensor is 9.53 mm. The area of the sensing area is calculated as $7.1331\times 10 ^{-5}\text{m}^{2}$ ( $A_{r}=\pi r^{2}$). More details on the strain tensor and the computation of the stress can be found in [6]. Briefly, the applied stress in units of [Pa] is calculated using $\sigma _{0}=\frac {F_{r}\times 4.4}{255\times A_{r}}$, where $F_{r}$ is the force reading obtained from the sensor during the experiments. It is noted that $\sigma _{0}$ is the axial component of the applied stress and the other two components (lateral and elevational) are assumed to be zero.

Ultrasound radio-frequency (RF) data acquisition was synchronized with the compression. Axial and lateral strains were computed from RF data using DPHS and Kalman filtering based method proposed in [33]. The tumor was segmented from the axial strain elastograms using Matlab, Mathworks Inc., Natick, MA, USA.

To compute VP inside the tumor, we need surface area to volume ratio of the capillary walls $\frac {S}{V}$ inside the tumor. This $\frac {S}{V}$ ratio was computed as [43]

\begin{equation*} \frac {S}{V}=10fV_{t}^{g}\end{equation*}

where $V_{t}$ is the volume of the tumor, $f =54.68$, $g = -0.2021$ [43]. Volume of the tumor is computed as $V_{t} =(4/3)\pi l_{1}^{2} l_{2} $. Because the tumor is approximated by its best fit ellipsoid, $l_{1}$ and $l_{2}$ are the minor and major axis lengths of the fitted ellipsoid, respectively. The third dimension of the ellipsoid is assumed to be equal to the minor axis length $l_{1}$. Here, $l_{1}$ and $l_{2}$ are in units of mm and $\frac {S}{V}$ is in units of cm^−1^.

## V. Results

### A. FE Simulation

**FIGURE 3.**
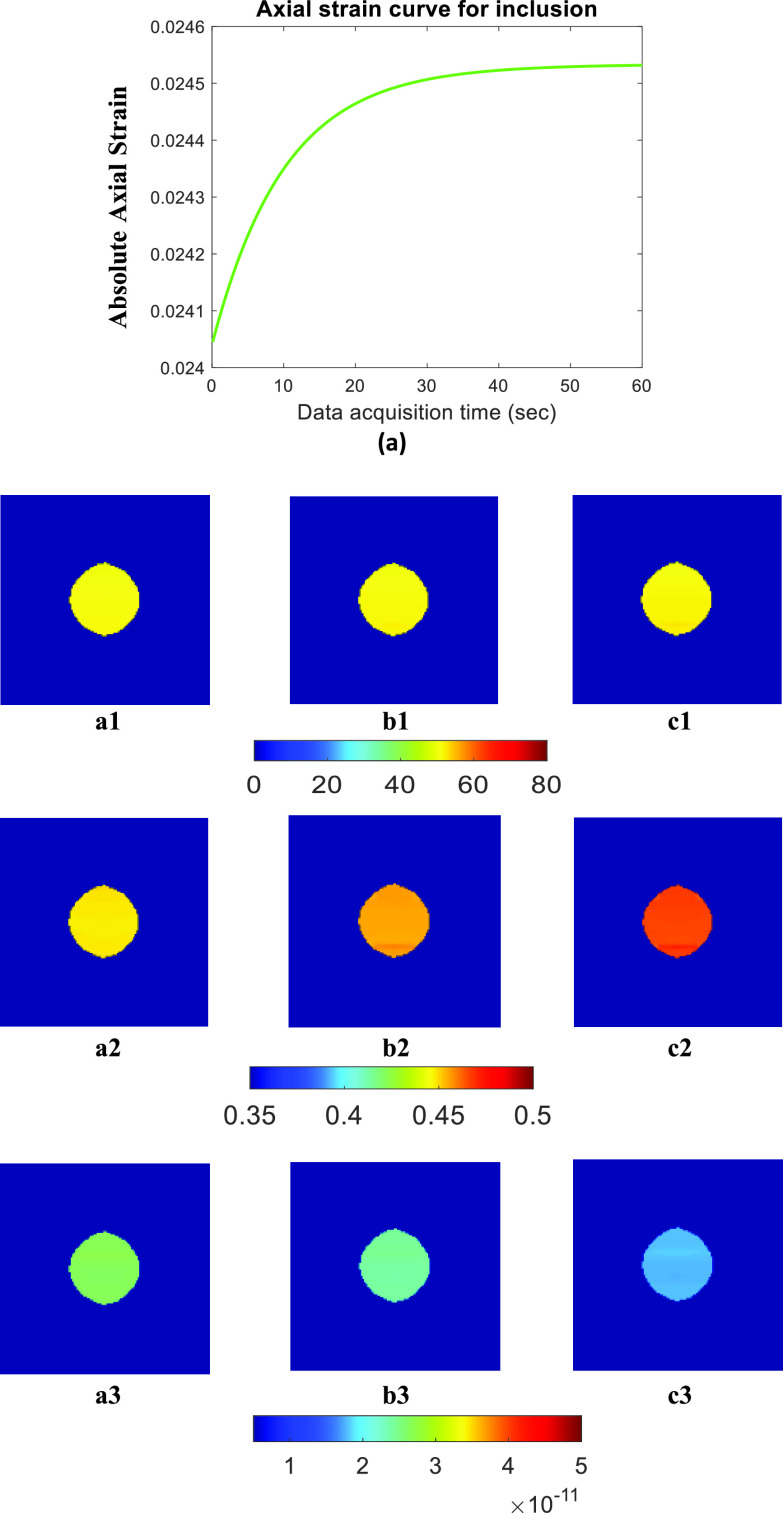
FE simulation data for sample A: (a) Axial strain curve vs. data acquisition time inside the inclusion. Estimated parameters from FEM data (1) YM (in kPa) (2) PR (3) VP (m(Pa $\cdot \text{s}$)^−1^) by using (A) full duration, (B) 2 TC, and (C) 1 TC WoOs. In this figure, the background is masked.

Fig. 3 and Fig. 5 show the ideal FE results obtained for Sample A (spherical inclusion) and Sample E (elliptical inclusion), respectively. In these figures, we show the reconstructed YM, PR, and VP obtained from the full duration window (60 s) as well as for shorter WoOs (0.5 TC, 1 TC, and 2 TC). The reconstructed parameters are shown for the inclusion only. From the FE results, the images of all 3 reconstructed parameters using WoO = 2 TC are qualitatively very similar to the corresponding images obtained using the steady state strains. For WoO = 1 TC, YM/ PR maps are also visually similar to the corresponding maps of YM/PR computed from steady state value. In the case of VP, if we consider the uniform region inside the inclusion, the VP value is also very similar to the VP estimated from the steady state data.

**FIGURE 4.**
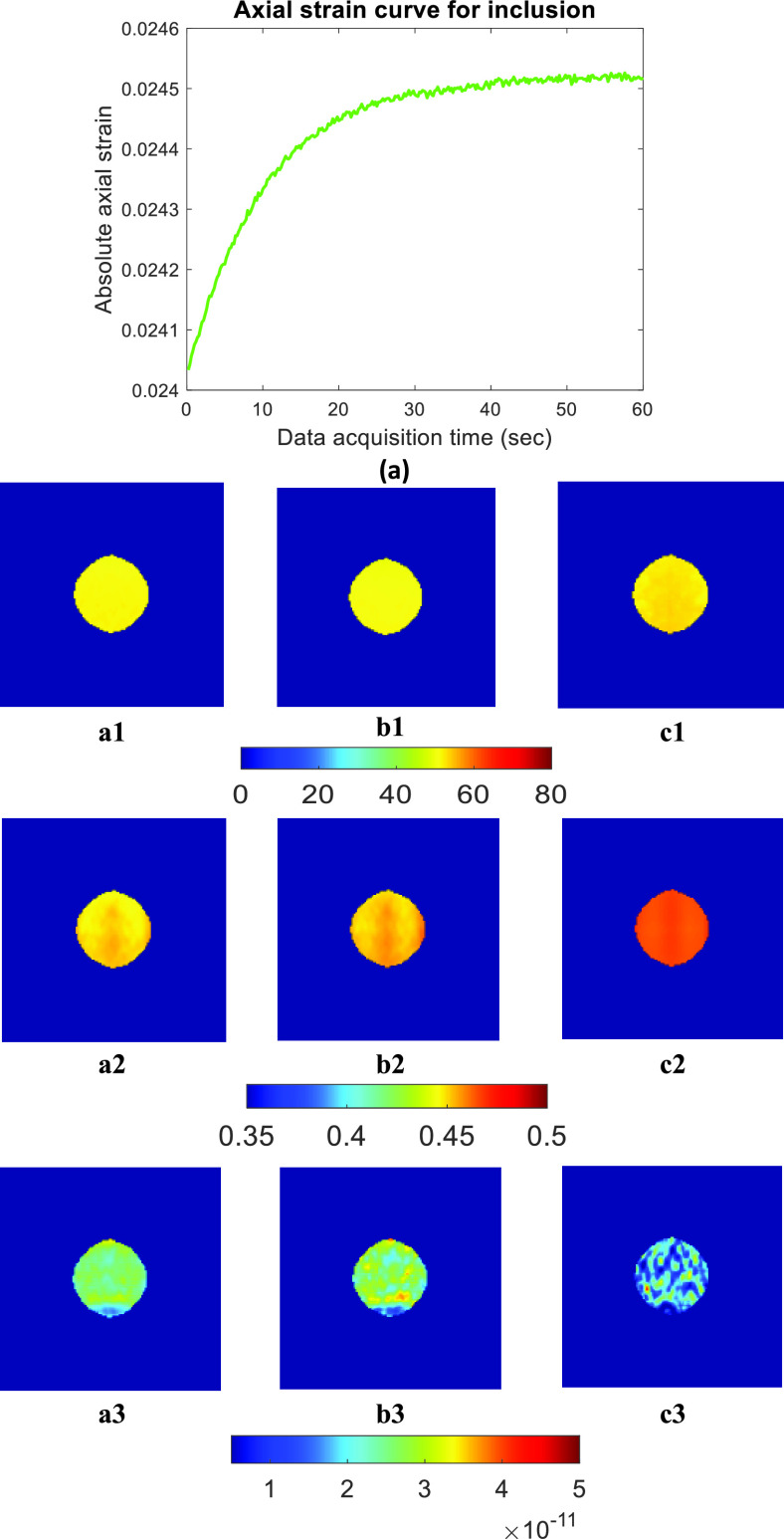
Ultrasound simulation data using 40 dB SNR for sample A: (a) Axial strain curve vs. data acquisition time inside the inclusion. Estimated parameters (1) YM (in kPa) (2) PR (3) VP (m(Pa $\cdot \text{s}$)^−1^) from ultrasound simulation by using (A) full duration, (B) 2 TC, and (C) 1 TC WoOs.

**FIGURE 5.**
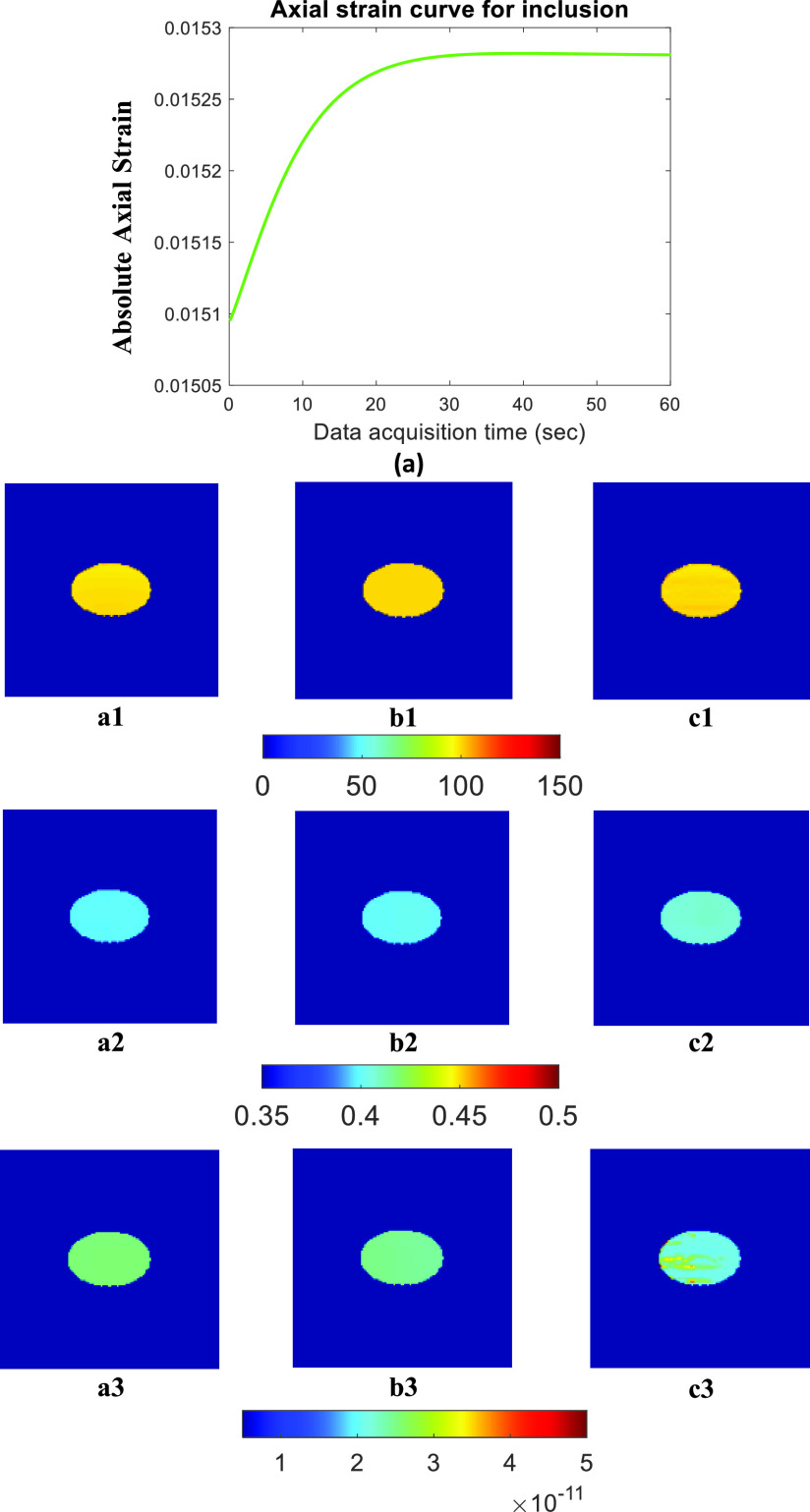
FE simulation data for sample E: (a) Axial strain curve vs. data acquisition time inside the inclusion. Estimated parameters from FEM data (1) YM (in kPa) (2) PR (3) VP (m(Pa $\cdot \text{s}$)^−1^) by using (A) full duration, (B) 2 TC, and (C) 1 TC WoOs. In this figure, the background is masked.

In Table 2, we report the PRE for the mechanical parameters (YM, PR) in all simulated samples. In Table 3, we report the PRE for the transport parameter (VP) in all simulated samples. We observe that, in general, the error increases as the WoO decreases. For all samples, error in YM estimation is < 1% for WoO= 2 TC, < 5% for WoO= 1 TC, and < 12% for WoO= 0.5 TC. In the case of PR estimation, PRE is < 4% for WoO= 2 TC, < 10% for WoO= 1 TC, and almost 15% for WoO= 0.5 TC. These results validate the theory detailed in the *Problem formulation* section of the paper.

**TABLE 2:** PRE (%) in Estimated YM, PR and one Standard Deviation (SD) of Ideal Value at 0.5 TC, 1 TC, and 2 TC Long Data

Sample	YM (kPa)						PR					
	2 TC		1 TC		0.5 TC		2 TC		1 TC		0.5 TC	
	PRE	Actual ± SD	PRE	Actual ± SD	PRE	Actual ± SD	PRE	Actual ± SD	PRE	Actual ± SD	PRE	Actual ± SD
A	0.89	50.00± 1.17	4.58	50.00± 1.53	11.22	50.00± 5.36	1.64	0.450± 0.005	4.09	0.450± 0.016	5.18	0.450± 0.163
B	0.27	97.02±4.37	0.87	97.02±4.94	6.87	97.02±6.54	1.13	0.450± 0.015	3.26	0.450± 0.021	4.96	0.450± 0.110
C	0.97	97.02±4.19	2.52	97.02±6.67	8.46	97.02±7.85	3.40	0.400 ± 0.017	7.54	0.400± 0.030	12.67	0.400± 0.182
D	0.21	97.02±3.80	0.61	97.02±4.18	5.58	97.02±6.29	0.76	0.470±0.002	2.17	0.470± 0.009	4.21	0.470± 0.080
E	0.93	97.02±4.80	4.17	97.02±3.03	10.59	97.02±7.13	2.85	0.400±0.036	9.61	0.400± 0.009	15.23	0.400± 0.090
F	0.29	50.00± 0.77	0.82	50.00± 1.06	5.45	50.00± 4.26	1.38	0.450±0.005	1.56	0.450± 0.014	3.54	0.450± 0.021

**TABLE 3:** PRE (%) in Estimated VP and One Standard Deviation (SD) of Ideal Value at 0.5 TC, 1 TC, and 2 TC Long Data

WoOs	VP (10 $^{\mathbf {-11}}\text{m}$(Pas) $^{\mathbf {-1}}$)											
	Sample A		Sample B		Sample C		Sample D		Sample E		Sample F	
	PRE	Actual ± SD	PRE	Actual ± SD	PRE	Actual ± SD	PRE	Actual ± SD	PRE	Actual ± SD	PRE	Actual ± SD
2 TC	6.21	2.820± 0.405	7.24	2.820± 0.822	6.92	2.820± 0.621	4.56	0.283± 0.059	6.55	2.820± 0.411	7.31	5.650 ± 0.521
1 TC	14.87	2.820± 1.141	21.74	2.820± 0.861	15.38	2.820± 1.194	11.95	0.283± 1.650	18.38	2.820± 1.520	17.29	5.650 ± 3.924
0.5 TC	45.5	2.820± 2.042	49.44	2.820± 2.860	53.64	2.820± 2.161	39.34	0.283± 3.150	59.35	2.820± 3.150	52.12	5.650 ± 5.740

In the case of VP, PRE is < 8% for WoO= 2 TC, < 20% for WoO= 1 TC. However, the error becomes large (>50%) in case of WoO= 0.5 TC. VP estimation requires accurate estimation of YM/PR and axial strain TC. The major contribution to this error comes from the strain TC estimation, which, in general, is significantly affected by the reduction of the WoOs. Our simulation results obtained for the axisymmetry model appear to confirm observations reported in a earlier publication [29]. We observe that, sample D, which has underlying TC 30.25s, has the lowest error in estimated VP. Overall, PRE in estimated VP is < 8% for WoO= 2 TC. Therefore, if reconstruction of transport parameters is required, WoOs of at least 2TC should be used. If the goal, instead, is reconstruction of the mechanical parameters (YM, PR) only, WoOs as low as 1 TC (or even lower, in some cases) may be considered.

### B. Ultrasound Simulations

**FIGURE 6.**
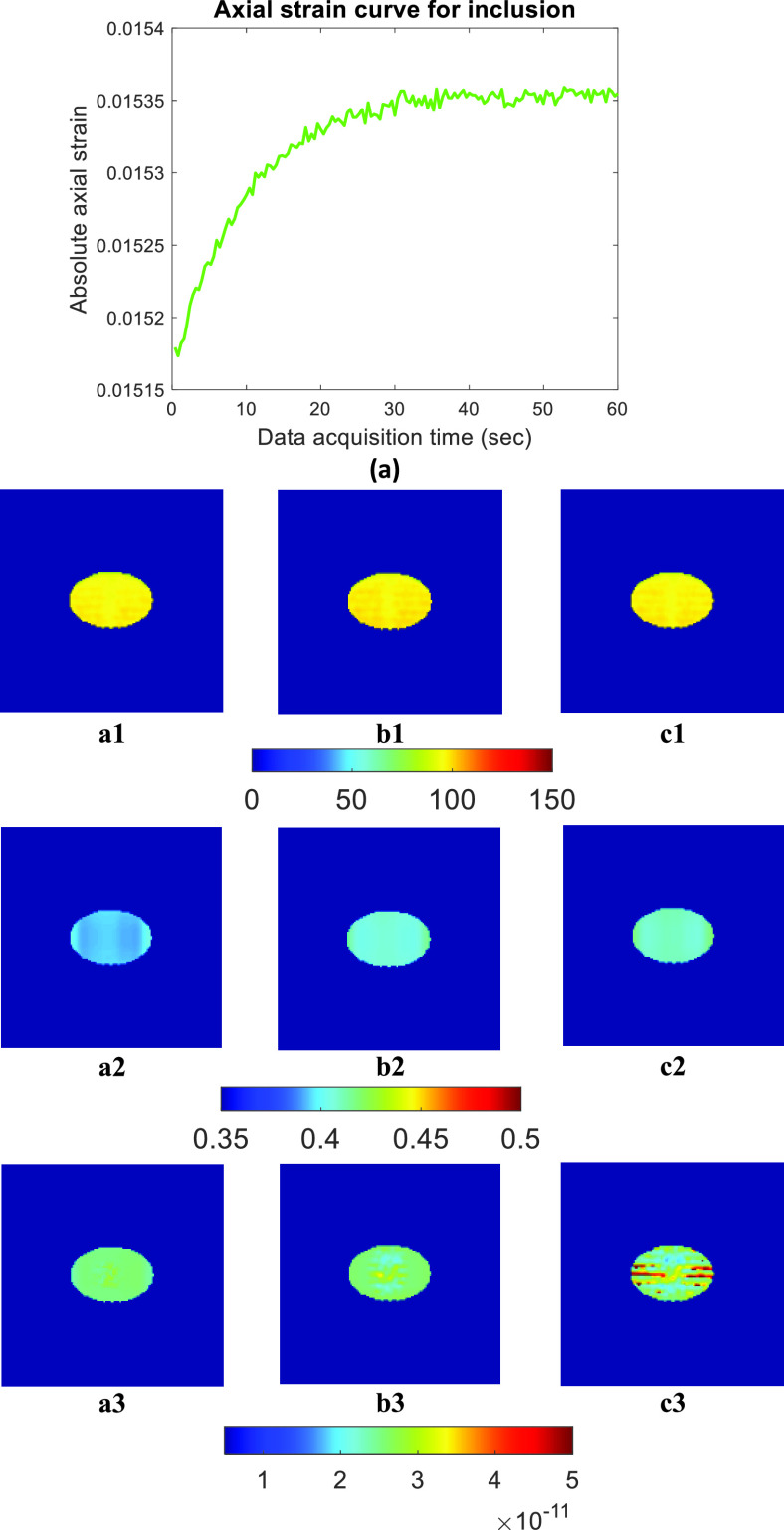
Ultrasound simulation data using 40 dB SNR for sample E: (a) Axial strain curve vs. data acquisition time inside the inclusion. Estimated parameters (1) YM (in kPa) (2) PR (3) VP (m(Pa $\cdot \text{s}$)^−1^) from ultrasound simulation by using (A) full duration, (B) 2 TC, and (C) 1 TC WoOs.

Fig. 4 and Fig. 6 show selected results from the ultrasound simulations (Sample A and Sample E, respectively for 40 dB SNR). With respect to the corresponding results in Fig. 3 and Fig. 5, these images are generally noisier (as ultrasound limitations and additive noise affect the results). Table 4 reports the PRE and SD in the estimated parameters in Sample A and Sample E for 40 dB and 20 dB SNR levels in case of WoO= 2 TC, WoO= 1 TC, and WoO= 0.5 TC. As for the FE results, the error increases with shorter WoOs. When WoO = 2 TC, reconstructed YM and PR have less than 5% PRE and VP has less than 8% PRE both for the 40 dB and the 20 dB SNR cases. Overall, the ultrasound simulation results are consistent with the FE findings.

**TABLE 4:** PRE (%) in Estimated YM, PR, and VP, and One Standard Deviation (SD) of Ideal Value at 0.5 TC, 1 TC, and 2 TC Long Data for 40 $dB$ and 20 $dB$ SNR Levels. Params.: Estimated Parameters. YM is in $kPa$. VP is in ( $10^{-11} m\left({PaS }\right))$

Sample Params.		40 dB SNR						20 dB SNR					
		2 TC		1 TC		0.5 TC		2 TC		1 TC		0.5 TC	
		PRE	Actual ± SD	PRE	Actual± SD	PRE	Actual ± SD	PRE	Actual ± SD	PRE	Actual ± SD	PRE	Actual ± SD
A	YM	1.09	50.00± 0.73	2.58	50.00± 2.60	4.22	50.00± 4.18	1.67	50.00± 1.69	2.79	50.00± 2.15	4.85	50.00± 3.26
	PR	0.84	0.450± 0.004	3.69	0.450± 0.030	6.28	0.450± 0.171	1.53	0.450± 0.004	4.04	0.450± 0.006	5.21	0.450± 0.009
	VP	4.09	2.82± 0.53	8.22	2.82± 1.78	14.15	2.82± 3.89	5.29	2.82± 0.54	8.45	2.82± 4.66	14.18	2.82± 6.25
E	YM	0.10	97.02± 2.20	0.74	97.02± 2.26	3.58	97.02± 5.15	1.84	97.02± 2.57	5.02	97.02± 6.21	7.17	97.02± 8.54
	PR	2.99	0.400± 0.006	5.008	0.400± 0.009	7.15	0.400± 0.024	5.03	0.400 ± 0.021	9.70	0.400±0.030	15.70	0.400 ± 0.081
	VP	4.92	2.82± 0.50	15.85	2.82± 2.93	43.39	2.82± 4.49	6.82	2.82± 0.83	28.02	2.82± 3.15	58.02	2.82± 4.58

### C. In Vivo Experiments

**FIGURE 7.**
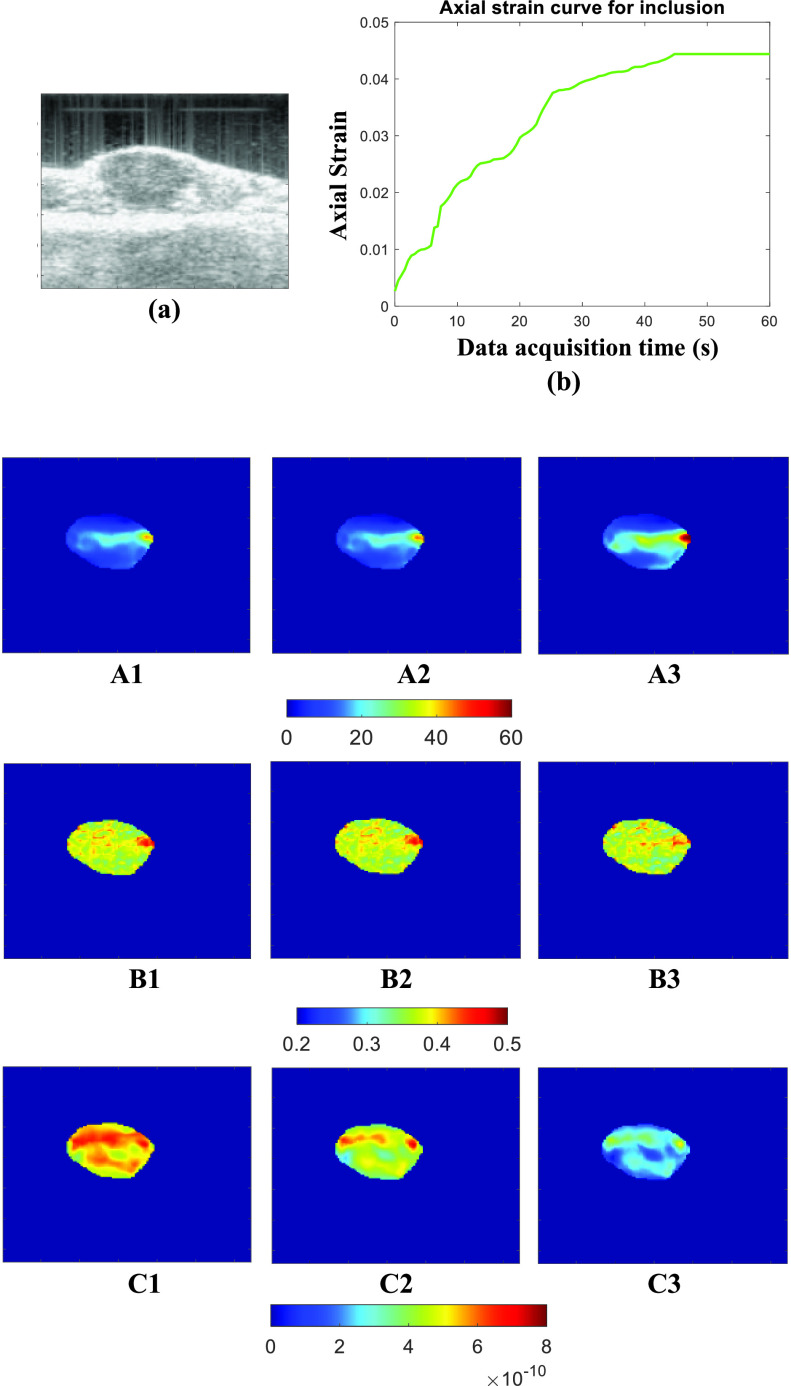
In vivo experiment 1:(a) Ultrasound B-mode image, (b) Axial strain curve vs. data acquisition time inside the inclusion. (A1-A3) YM estimated using full set of experimental data, WoO = 2 TC, and WoO= 1 TC. (B1-B3) PR estimated using full set of experimental data, WoO = 2 TC, and WoO= 1 TC. (C1-C3) VP estimated using full set of experimental data, WoO = 2 TC, and WoO= 1 TC, respectively.

**FIGURE 8.**
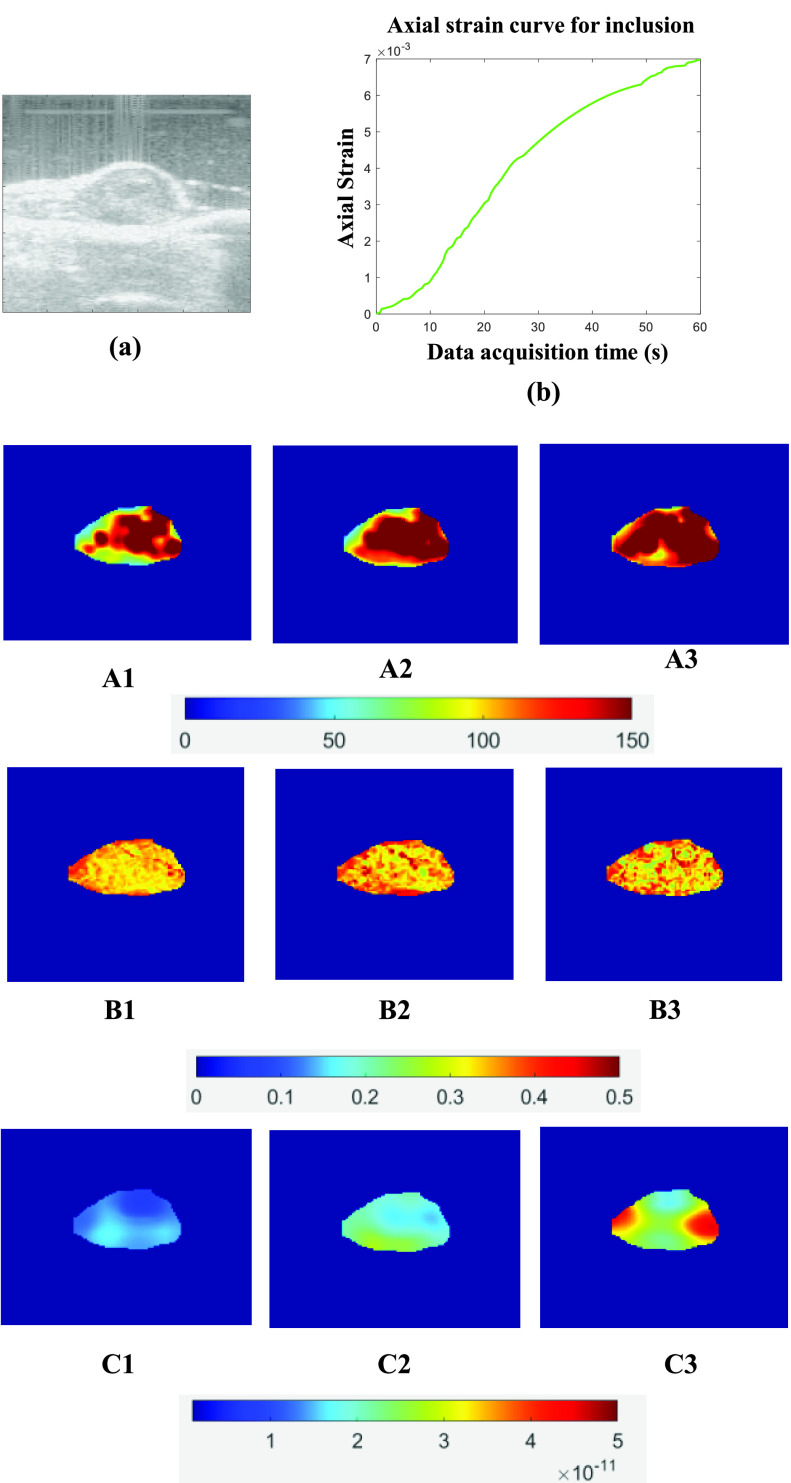
In vivo experiment 2:(a) Ultrasound B-mode image, (b) Axial strain curve vs. data acquisition time inside the inclusion. (A1-A3) YM estimated using full set of experimental data, WoO = 2 TC, and WoO= 1 TC. (B1-B3) PR estimated using full set of experimental data, WoO = 2 TC, and WoO= 1 TC. (C1-C3) VP estimated using full set of experimental data, WoO = 2 TC, and WoO= 1 TC, respectively.

Fig. 7 and Fig. 8 show the experimental results for two *in vivo* experiments. B-mode image, axial strain curve, and corresponding reconstructed YM, PR, and VP for different WoOs are shown in these figures. For these two *in vivo* data shown in Fig. 7 and Fig. 8, axial strain TC values are 17.58 sec and 21.26 sec, respectively. From both figures, we see that there is no significant change in YM and PR estimates when using WoO of at least 1 TC, as predicted by the theory. VP map of the tumor is affected by the use of short WoO (C1-C3). The PRE (%) values computed from estimated YM, PR and VP in *in vivo* cases are shown in Table 5. PRE for the estimated YM and PR is < 4% for WoO = 2 TC while VP PRE is < 11% for WoO = 2 TC. The recorded applied pressure was approximately 740 Pa for this *in vivo* data.

**TABLE 5:** PRE (%) in estimated YM, PR, VP and one standard deviation (SD) of mean value for two $in \,vivo$ experiments at different time points. YM is in $kPa$. VP is in ( $10^{-11} m\left({PaS }\right)^{-1})$

Reconstructed Parameters	Experiment 1				Experiment 2					
	2 TC		1 TC		2 TC		1 TC			
	PRE	Mean±SD	PRE	Mean±SD	PRE	Mean±SD	PRE	Mean±SD
YM	3.42	20.09± 7.63	16.18	25.09± 9.89	8.44	130.61± 15.03	41.53	180.58± 41.90
PR	0.59	0.39± 0.03	2.862	0.38± 0.03	2.91	0.35±0.04	9.21	0.34±0.04
VP	10.04	49.25± 6.21	29.41	37.89± 5.19	12.76	1.99± 0.30	71.95	8.10±2.00

## VI. Discussion

In this paper, we investigate the accuracy of YM, PR and VP reconstructions in spherical and elliptical inclusions when using poroelastography techniques with different temporal windows of observations. Using shorter windows of observations would facilitate *in vivo* applications of elasticity imaging methods.

In this study, we have focused on three fundamental tissue parameters. YM is one of the most important markers for cancer detection and cancer progression [6]. PR is another important mechanical property related to the compressibility of a tissue, whose role in cancers remains under-investigated but was found informative in cancer-related diseases such as lymphedema [27], [44]. VP is a parameter of great importance for diagnosis, prognosis and treatment of cancers [5], [40]. For the theoretical formulation of the problem, we assumed spherical/elliptical inclusions embedded in a cylindrical background [6], [30]. This choice is motivated by the fact that, in most literature, the tumor is assumed to be elliptical or spherical [5], [45], [46]. In addition, irregularly shaped cancers can be approximated with spheres or ellipsoids [30], [34], [35], [47], [48].

Overall, our results show that WoOs of at least 1 strain TC are sufficient to reconstruct the mechanical parameters (YM/PR) and at least 2 strain TC are sufficient for estimating transport parameters (VP) of tumor within 10% error. We validated our theoretical observations using FEM simulation and ultrasound simulation. In vivo data were used to further corroborate the theoretical and simulation findings when dealing with low SNR and practical acquisition frame rates.

A number of factors can affect the performance of the proposed method. The first one is the quality of the axial and lateral strains estimated from ultrasound poroelastography data. Lateral strain estimation is noisy due to pitch limitation and the baseband characteristics of the lateral signal. However, several approaches have been proposed to improve the image quality of lateral strain elastograms. Among these, our group has proposed a two-step strain estimation method referred to as “DPHS” consisting of Dynamic Programming Elastography (DPE) and Horn-Shunk (HS) method [33]. In a more recent paper [49],we have proposed a filtering technique combining Kalman filter and nonlinear complex diffusion filters (NCDF) to further reduce the additive and amplitude modulation noises in the estimated strains, which has been proven to be very effective for high quality strain estimations. Another important factor that can affect the performance of the proposed approach is the quality of the YM and PR estimates. To reconstruct YM and PR, we used the Eshelby’s theory proposed in [6].

Correct application of STPE still requires several seconds of data acquisition, time that is needed for the tissue to manifest its own intrinsic properties. This is true for any elasticity imaging technique, because it is inherent to the tissue mechanical response to the excitation. In practical cases, depending on the type of cancer or animal models, we can establish a range of strain TC values. For example, for the animal model used in our experiments, the strain TC was found to be in the range of 10-15 s [30], [37]. These acquisition lengths typically allow to accurately track the strains if strategies to keep the temporal frames correlated are employed (such as speckle tracking using closely spaced temporal frames, etc. [41]). In cases where the underlying strain TC is unknown, a preliminary prolonged data acquisition may still be required. However, successive experiments from the same tumor could be more efficiently performed using STPE. Even in the case of data acquisition from multiple planes, the total experimental time can still be significantly lower than the time required by other imaging modalities such as CT, MRI etc. Based on our previous experience, an elastography experiment of 10-15 s duration is feasible in clinics and our developed technique should help translation to the clinics.

Another important factor affecting the performance of the proposed method is the algorithm used to estimate VP. In Figs. (7) and (8), we can see that the reconstructed VP map is significantly different for 1 TC data than the VP estimated by full window data. We reconstructed VP using the method proposed in [30], which requires estimation of YM, PR, and axial strain TC. Errors associated with the estimated YM, PR, and axial strain TC leads to a combined larger error for VP. The algorithm used for estimating VP is based on the assumption of dominance of VP over IP inside the tumors and normal tissues. This assumption is typically valid for cancers [5], [45], [50], [51] as most fluid flow occurs through the capillary walls. Therefore, VP and axial strain TC in cancers should not be spatially variant inside the inclusion [28], [52].

Finally, we acknowledge the limited *in vivo* experiment results presented in this paper. These results are shown as a proof-of-principle of the potential applicability of the proposed STPE method in practical scenario. Additional experiments are required to further corroborate the use STPE in different experimental scenarios.

## VII. Conclusion

In this paper, we designed and analyzed a non-invasive method to image the YM, PR and VP of tumors using short duration ultrasound poroelastography experiments. Simulations proved that the proposed technique allows estimation of the mechanical parameters with high accuracy. Transport parameters, such as VP, can also be accurately reconstructed using reduced windows of observations but require longer acquisitions than YM and PR. The proposed method could facilitate *in vivo* applications of cancer poroelastography.
